# Supplementary material for: Vulnerability of agriculture to climate change increases the risk of child malnutrition: Evidence from a large-scale observational study in India
Source: PLoS One. 2021 Jun 28;16(6):e0253637. doi: 10.1371/journal.pone.0253637 (PMC8238181; doi:10.1371/journal.pone.0253637)
Supplement: S1 Table — (DOCX) [file pone.0253637.s002.docx]

**S1 Table:** **Districts categorized as “high” or “very high” in degree of vulnerability and having child malnutrition levels higher than India average**

|  | **Stunting** | **Wasting** | **Underweight** | **Anaemia** |
| --- | --- | --- | --- | --- |
| Agra (UP) | 🗸 |  |  |  |
| Ahmadabad (GJ) |  | 🗸 |  | 🗸 |
| Ahmadnagar (MH) |  | 🗸 |  |  |
| Ajmer (RJ) |  | 🗸 | 🗸 | 🗸 |
| Akola (MH) | 🗸 | 🗸 | 🗸 |  |
| Alirajpur (MP) | 🗸 | 🗸 | 🗸 | 🗸 |
| Alwar (RJ) | 🗸 |  |  |  |
| Amravati (MH) |  | 🗸 |  |  |
| Amreli (GJ) |  | 🗸 |  | 🗸 |
| Anantapur (AP) | 🗸 |  | 🗸 |  |
| Anuppur (MP) |  | 🗸 | 🗸 | 🗸 |
| Araria (BR) | 🗸 | 🗸 | 🗸 | 🗸 |
| Ashoknagar (MP) | 🗸 | 🗸 | 🗸 | 🗸 |
| Aurangabad (MH) |  |  | 🗸 |  |
| Bagalkot (KN) | 🗸 | 🗸 | 🗸 | 🗸 |
| Bageshwar (UK) |  | 🗸 |  |  |
| Baghpat (UP) |  |  |  | 🗸 |
| Bahraich (UP) | 🗸 |  | 🗸 | 🗸 |
| Ballia (UP) | 🗸 |  |  | 🗸 |
| Balrampur (UP) | 🗸 |  | 🗸 | 🗸 |
| Banas Kantha (GJ) | 🗸 | 🗸 | 🗸 |  |
| Banda (UP) | 🗸 |  | 🗸 | 🗸 |
| Bangalore Rural (KN) |  | 🗸 |  |  |
| Banswara (RJ) | 🗸 | 🗸 | 🗸 | 🗸 |
| Baran (RJ) | 🗸 | 🗸 | 🗸 | 🗸 |
| Barmer (RJ) |  | 🗸 | 🗸 | 🗸 |
| Barpeta (AS) | 🗸 |  |  |  |
| Barwani (MP) | 🗸 | 🗸 | 🗸 | 🗸 |
| Basti (UP) | 🗸 |  |  | 🗸 |
| Belgaum (KN) |  | 🗸 | 🗸 | 🗸 |
| Bellary (KN) | 🗸 | 🗸 | 🗸 | 🗸 |
| Betul (MP) |  | 🗸 | 🗸 | 🗸 |
| Bhagalpur (BR) | 🗸 | 🗸 | 🗸 | 🗸 |
| Bharatpur (RJ) | 🗸 |  |  |  |
| Bharuch (GJ) | 🗸 | 🗸 | 🗸 |  |
| Bhavnagar (GJ) | 🗸 | 🗸 | 🗸 | 🗸 |
| Bhilwara (RJ) |  | 🗸 | 🗸 | 🗸 |
| Bhind (MP) | 🗸 | 🗸 | 🗸 | 🗸 |
| Bhiwani (HR) |  |  |  | 🗸 |
| Bid (MH) |  | 🗸 | 🗸 |  |
| Bidar (KN) | 🗸 | 🗸 | 🗸 | 🗸 |
| Bijapur (CH) | 🗸 | 🗸 | 🗸 |  |
| Bijapur (KN) | 🗸 | 🗸 | 🗸 | 🗸 |
| Bikaner (RJ) |  | 🗸 |  |  |
| Bokaro (JH) | 🗸 | 🗸 | 🗸 | 🗸 |
| Budaun (UP) | 🗸 |  | 🗸 |  |
| Buldana (MH) | 🗸 | 🗸 | 🗸 |  |
| Bundi (RJ) |  | 🗸 | 🗸 | 🗸 |
| Buxar (BR) | 🗸 |  | 🗸 | 🗸 |
| Chamba (HP) |  |  |  | 🗸 |
| Chhatarpur (MP) | 🗸 |  | 🗸 | 🗸 |
| Chhindwara (MP) |  | 🗸 | 🗸 | 🗸 |
| Chikkaballapura (KN) |  |  |  | 🗸 |
| Chitradurga (KN) |  | 🗸 |  | 🗸 |
| Chitrakoot (UP) | 🗸 | 🗸 | 🗸 | 🗸 |
| Chittaurgarh (RJ) |  | 🗸 | 🗸 | 🗸 |
| Churu (RJ) |  | 🗸 |  |  |
| Dakshin Bastar Dantewada (CH) | 🗸 | 🗸 | 🗸 | 🗸 |
| Damoh (MP) | 🗸 |  | 🗸 | 🗸 |
| Darbhanga (BR) | 🗸 |  | 🗸 | 🗸 |
| Datia (MP) | 🗸 | 🗸 | 🗸 | 🗸 |
| Davanagere (KN) | 🗸 | 🗸 | 🗸 | 🗸 |
| Deoria (UP) | 🗸 |  |  | 🗸 |
| Dewas (MP) | 🗸 | 🗸 | 🗸 | 🗸 |
| Dhar (MP) | 🗸 | 🗸 | 🗸 | 🗸 |
| Dharmapuri (TN) |  | 🗸 |  |  |
| Dharwad (KN) |  | 🗸 | 🗸 |  |
| Dhaulpur (RJ) | 🗸 |  | 🗸 |  |
| Dhule (MH) | 🗸 | 🗸 | 🗸 | 🗸 |
| Dindigul (TN) |  | 🗸 |  |  |
| Dindori (MP) | 🗸 | 🗸 | 🗸 | 🗸 |
| Dohad (GJ) | 🗸 | 🗸 | 🗸 |  |
| Dungarpur (RJ) | 🗸 | 🗸 | 🗸 | 🗸 |
| Durg (CH) |  | 🗸 | 🗸 |  |
| Faizabad (UP) | 🗸 |  | 🗸 | 🗸 |
| Faridkot (PB) |  | 🗸 |  | 🗸 |
| Fatehabad (HR) |  |  |  | 🗸 |
| Fatehpur (UP) | 🗸 |  | 🗸 |  |
| Firozpur (PB) |  | 🗸 |  |  |
| Gadag (KN) |  | 🗸 | 🗸 | 🗸 |
| Gandhinagar (GJ) |  | 🗸 | 🗸 | 🗸 |
| Ghazipur (UP) | 🗸 |  |  | 🗸 |
| Godda (JH) | 🗸 | 🗸 | 🗸 | 🗸 |
| Gonda (UP) | 🗸 |  | 🗸 | 🗸 |
| Gopalganj (BR) |  |  |  | 🗸 |
| Gulbarga (KN) | 🗸 | 🗸 | 🗸 | 🗸 |
| Gumla (JH) | 🗸 | 🗸 | 🗸 | 🗸 |
| Guna (MP) | 🗸 | 🗸 | 🗸 | 🗸 |
| Hamirpur (UP) |  | 🗸 | 🗸 |  |
| Haveri (KN) | 🗸 |  | 🗸 | 🗸 |
| Hisar (HR) |  | 🗸 |  | 🗸 |
| Jaisalmer (RJ) |  | 🗸 | 🗸 |  |
| Jalaun (UP) | 🗸 | 🗸 | 🗸 | 🗸 |
| Jalgaon (MH) |  | 🗸 | 🗸 | 🗸 |
| Jalna (MH) | 🗸 | 🗸 | 🗸 |  |
| Jalor (RJ) | 🗸 | 🗸 | 🗸 | 🗸 |
| Jamnagar (GJ) |  | 🗸 |  | 🗸 |
| Jaunpur (UP) | 🗸 | 🗸 | 🗸 |  |
| Jhabua (MP) | 🗸 | 🗸 | 🗸 | 🗸 |
| Jhajjar (HR) |  |  |  | 🗸 |
| Jhalawar (RJ) |  | 🗸 | 🗸 | 🗸 |
| Jhansi (UP) |  | 🗸 | 🗸 | 🗸 |
| Jind (HR) |  | 🗸 |  | 🗸 |
| Jodhpur (RJ) | 🗸 | 🗸 | 🗸 | 🗸 |
| Junagadh (GJ) |  | 🗸 |  | 🗸 |
| Kabeerdham (CH) | 🗸 |  | 🗸 |  |
| Kachchh (GJ) | 🗸 | 🗸 | 🗸 | 🗸 |
| Kaithal (HR) |  | 🗸 | 🗸 | 🗸 |
| Kapurthala (PB) |  |  |  | 🗸 |
| Karauli (RJ) | 🗸 |  |  |  |
| Karur (TN) |  | 🗸 |  |  |
| Katihar (BR) | 🗸 |  | 🗸 | 🗸 |
| Katni (MP) | 🗸 | 🗸 | 🗸 | 🗸 |
| Kaushambi (UP) | 🗸 | 🗸 | 🗸 | 🗸 |
| Kishanganj (BR) | 🗸 | 🗸 | 🗸 | 🗸 |
| Kishtwar (JK) |  |  |  | 🗸 |
| Koppal (KN) | 🗸 | 🗸 | 🗸 | 🗸 |
| Koriya (CH) |  | 🗸 |  |  |
| Kurnool (AP) | 🗸 |  | 🗸 |  |
| Kushinagar (UP) | 🗸 |  |  |  |
| Lalitpur (UP) | 🗸 | 🗸 | 🗸 | 🗸 |
| Latehar (JH) | 🗸 | 🗸 | 🗸 |  |
| Latur (MH) |  | 🗸 |  |  |
| Lohardaga (JH) | 🗸 | 🗸 | 🗸 | 🗸 |
| Madhubani (BR) | 🗸 |  | 🗸 | 🗸 |
| Maharajganj (UP) | 🗸 |  | 🗸 |  |
| Mahbubnagar (AP) |  |  |  | 🗸 |
| Mahendragarh (HR) |  |  |  | 🗸 |
| Mahesana (GJ) | 🗸 | 🗸 | 🗸 | 🗸 |
| Mahoba (UP) | 🗸 | 🗸 | 🗸 | 🗸 |
| Maldah (WB) |  | 🗸 | 🗸 |  |
| Mandla (MP) |  | 🗸 | 🗸 | 🗸 |
| Mandsaur (MP) |  | 🗸 |  | 🗸 |
| Mandya (KN) |  | 🗸 |  |  |
| Mathura (UP) | 🗸 |  |  |  |
| Mau (UP) | 🗸 |  |  | 🗸 |
| Mirzapur (UP) | 🗸 |  | 🗸 | 🗸 |
| Muzaffarpur (BR) | 🗸 |  | 🗸 |  |
| Mysore (KN) |  |  |  | 🗸 |
| Nagaur (RJ) | 🗸 |  |  |  |
| Nalanda (BR) | 🗸 | 🗸 | 🗸 |  |
| Nandurbar (MH) | 🗸 | 🗸 | 🗸 | 🗸 |
| Narmada (GJ) | 🗸 | 🗸 | 🗸 |  |
| Nashik (MH) | 🗸 | 🗸 | 🗸 |  |
| Neemuch (MP) |  | 🗸 | 🗸 | 🗸 |
| Nuapada (OD) |  | 🗸 | 🗸 | 🗸 |
| Osmanabad (MH) | 🗸 | 🗸 | 🗸 |  |
| Pakur (JH) | 🗸 | 🗸 | 🗸 | 🗸 |
| Palamu (JH) | 🗸 | 🗸 | 🗸 | 🗸 |
| Pali (RJ) | 🗸 | 🗸 | 🗸 |  |
| Panch Mahals (GJ) | 🗸 | 🗸 | 🗸 |  |
| Panipat (HR) | 🗸 | 🗸 | 🗸 | 🗸 |
| Panna (MP) | 🗸 | 🗸 | 🗸 | 🗸 |
| Parbhani (MH) | 🗸 |  | 🗸 |  |
| Pashchim Champaran (BR) | 🗸 | 🗸 | 🗸 | 🗸 |
| Pashchimi Singhbhum (JH) | 🗸 | 🗸 | 🗸 | 🗸 |
| Patan (GJ) |  | 🗸 | 🗸 | 🗸 |
| Patna (BR) | 🗸 | 🗸 | 🗸 |  |
| Porbandar (GJ) |  | 🗸 |  | 🗸 |
| Pratapgarh (RJ) | 🗸 | 🗸 | 🗸 | 🗸 |
| Purba Champaran (BR) | 🗸 |  | 🗸 | 🗸 |
| Purbi Singhbhum (JH) | 🗸 | 🗸 | 🗸 | 🗸 |
| Purnia (BR) | 🗸 |  | 🗸 | 🗸 |
| Rae Bareli (UP) |  | 🗸 | 🗸 | 🗸 |
| Raichur (KN) |  | 🗸 | 🗸 | 🗸 |
| Rajgarh (MP) |  | 🗸 | 🗸 | 🗸 |
| Rajkot (GJ) |  | 🗸 |  |  |
| Rajnandgaon (CH) | 🗸 |  | 🗸 |  |
| Rajsamand (RJ) |  | 🗸 | 🗸 | 🗸 |
| Ratlam (MP) | 🗸 | 🗸 | 🗸 | 🗸 |
| Rewa (MP) | 🗸 |  | 🗸 |  |
| Sabar Kantha (GJ) | 🗸 | 🗸 | 🗸 | 🗸 |
| Saharsa (BR) | 🗸 | 🗸 | 🗸 | 🗸 |
| Sahibganj (JH) | 🗸 | 🗸 | 🗸 | 🗸 |
| Salem (TN) |  | 🗸 |  |  |
| Samastipur (BR) | 🗸 |  | 🗸 | 🗸 |
| Saraikela-kharsawan (JH) | 🗸 | 🗸 | 🗸 | 🗸 |
| Saran (chhapra) (BR) | 🗸 |  | 🗸 | 🗸 |
| Satna (MP) | 🗸 | 🗸 | 🗸 | 🗸 |
| Sawai Madhopur (RJ) | 🗸 |  |  |  |
| Sehore (MP) |  | 🗸 | 🗸 | 🗸 |
| Shahdol (MP) |  | 🗸 | 🗸 | 🗸 |
| Shahjahanpur (UP) | 🗸 | 🗸 | 🗸 | 🗸 |
| Shajapur (MP) | 🗸 | 🗸 | 🗸 | 🗸 |
| Shimla (HP) |  |  |  | 🗸 |
| Shivpuri (MP) | 🗸 | 🗸 | 🗸 | 🗸 |
| Shrawasti (UP) | 🗸 |  | 🗸 | 🗸 |
| Siddharth Nagar (UP) | 🗸 |  | 🗸 | 🗸 |
| Sidhi (MP) | 🗸 | 🗸 | 🗸 | 🗸 |
| Simdega (JH) | 🗸 | 🗸 | 🗸 | 🗸 |
| Singrauli (MP) |  | 🗸 | 🗸 | 🗸 |
| Sirohi (RJ) | 🗸 | 🗸 | 🗸 | 🗸 |
| Sirsa (HR) |  | 🗸 |  | 🗸 |
| Sitamarhi (BR) | 🗸 |  | 🗸 | 🗸 |
| Siwan (BR) |  |  |  | 🗸 |
| Solapur (MH) |  | 🗸 |  |  |
| Sonbhadra (UP) | 🗸 | 🗸 | 🗸 |  |
| Supaul (BR) | 🗸 |  | 🗸 | 🗸 |
| Surendranagar (GJ) | 🗸 | 🗸 | 🗸 | 🗸 |
| Surguja (CH) |  | 🗸 |  |  |
| Tehri Garhwal (UK) |  | 🗸 | 🗸 | 🗸 |
| The Dangs (GJ) | 🗸 | 🗸 | 🗸 | 🗸 |
| Thiruvarur (TN) |  | 🗸 |  |  |
| Tiruvannamalai (TN) |  | 🗸 |  |  |
| Tonk (RJ) |  | 🗸 | 🗸 | 🗸 |
| Tumkur (KN) |  | 🗸 |  |  |
| Udaipur (RJ) | 🗸 | 🗸 | 🗸 | 🗸 |
| Ujjain (MP) |  |  |  | 🗸 |
| Umaria (MP) | 🗸 | 🗸 | 🗸 | 🗸 |
| Vadodara (GJ) | 🗸 |  | 🗸 |  |
| Vaishali (BR) | 🗸 |  | 🗸 | 🗸 |
| Vidisha (MP) | 🗸 | 🗸 | 🗸 | 🗸 |
| Washim (MH) | 🗸 | 🗸 | 🗸 | 🗸 |
| West Nimar (MP) | 🗸 | 🗸 | 🗸 | 🗸 |
| Yadgir (KN) | 🗸 | 🗸 | 🗸 | 🗸 |

**Colour legend for Column 1:**

|  | District has all four forms of malnutrition |
| --- | --- |
|  | District has three forms of malnutrition |
|  | District has two forms of malnutrition |
|  | District has only one form of malnutrition |
