# Supplementary material for: Vulnerability of agriculture to climate change increases the risk of child malnutrition: Evidence from a large-scale observational study in India
Source: PLoS One. 2021 Jun 28;16(6):e0253637. doi: 10.1371/journal.pone.0253637 (PMC8238181; doi:10.1371/journal.pone.0253637)
Supplement: Attachment — Submitted filename: Response to reviewer comments.docx [file pone.0253637.s003.docx]

**PONE-D-21-00139: Vulnerability of agriculture to climate change increases the risk of child malnutrition: Evidence from a large-scale observational study in India**

Dear Dr. Goli,

Academic Editor

PLOS ONE

Thank you for giving us an opportunity to strengthen the paper by addressing suggestions from you and reviewers. We have made the necessary changes to the manuscript. Following is our response to the comments.

**Editor’s comments**

Considering that weather stations to measure different climatic indicators are not present in the every district, rather they are very few in rural areas. Thus, researchers depend on simulation and extrapolation methods to derive the indicators at the district level. In this process, there is a chance of errors that need to be examined and reported under the limitation section. I request you to add a limitation section for the paper and validate climatic data.

Response: Many thanks for bringing this to our attention. We have included the non-availability of climatic indicators in every district as a limitation of the vulnerability index under limitation section.

**Journal Requirements:**

1. Please ensure that your manuscript meets PLOS ONE's style requirements, including those for file naming. The PLOS ONE style templates can be found at <https://journals.plos.org/plosone/s/file?id=wjVg/PLOSOne_formatting_sample_main_body.pdf> and

<https://journals.plos.org/plosone/s/file?id=ba62/PLOSOne_formatting_sample_title_authors_affiliations.pdf>

Response: We have reviewed the journal guidelines and have made corrections wherever it was required.

2. Please add details of how others can access the climate dataset in the Data availability statement.

Response: The climate data can be accessed by giving request to Indian Meteorological Department at <http://dsp.imdpune.gov.in/>

3. We note that Figure S1 and Figure 2 in your submission contain map images which may be copyrighted. All PLOS content is published under the Creative Commons Attribution License (CC BY 4.0), which means that the manuscript, images, and Supporting Information files will be freely available online, and any third party is permitted to access, download, copy, distribute, and use these materials in any way, even commercially, with proper attribution. For these reasons, we cannot publish previously copyrighted maps or satellite images created using proprietary data, such as Google software (Google Maps, Street View, and Earth). For more information, see our copyright guidelines: <http://journals.plos.org/plosone/s/licenses-and-copyright>.

We require you to either (1) present written permission from the copyright holder to publish these figures specifically under the CC BY 4.0 license, or (2) remove the figures from your submission. You may seek permission from the original copyright holder of Figure S1 and Figure 2 to publish the content specifically under the CC BY 4.0 license.

Response: The figures are authors’ own work; hence, we don’t need to take any permission. These maps were created using shape files which were made available by DataMet community projects map under CC BY 4.0 license (<https://github.com/datameet/maps/commit/2a00aae131ddbc25cbf43667c94c7416bc667dd3>).

**Reviewers' comments:**

**Reviewer #1:**

1. L 32-33....the aim of this study that was written in the introduction is not acceptable, the global warming treat negatively affects human nutrition all over the world not only in India. This is not the first study to examine such a relationship; however, the measuring tools and the data analyses style might provide an advantage for this research over many others. I think authors should re-write this aim.

Response: We agree that our study is not the first one in India to study the negative effects of global warming, however, as also highlighted by Reviewer #2, it is the first of its kind wherein relationship between vulnerability of agriculture to climate change and child nutrition in India using vulnerability index has been studied. We have changed the text in introduction section of the abstract to as per the suggestion.

2. The second point is related to the measuring tools that are used in this study. I think they should be rewritten in details.

Response: As suggested, we have provided more details about the measurement process and indicators. Accordingly, we have moved the supplementary table on indicators (Table S1) as a main table (Table 1) in the revised paper.

**Reviewer #2:**

1. Rewrite introduction. Brin first sentence (line no 302-04) to the begining of introduction. \

Line 53, introduction, avoid giving magnitude by 2080- which is very long. Put till 2050. line 60, avoid 10-60% give number

Response: Thank you for your suggestion on rewriting introduction. As suggested, we have moved first line from discussion section to the beginning of introduction. Regarding, providing the estimate till year 2050 instead of 2080, as climatic changes take longer to reflect and transform, the models and predictions studying the impact over next 50 years or more tend to be more reliable. Also, since 2080 is only 59 years away, we feel the estimates for 2080 aren’t very far off in future.

2. Para on India in intro and para on India in discussion can be combined and placed after literature review in intro.

Response: After reviewing the suggestion by the reviewer, we feel the para on India is better suited in discussion. Therefore, we have retained the paragraph on India in the discussion as it summarizes the situation in national context well and establishes a linkage between vulnerability of agriculture to climate change and child malnutrition.

3.Review, 13-17, give finding and not listing studies

Response: Thank you for the suggestion. We have added summary of findings from the studies cited in reference 13-17 in the introduction.

4.Data source: The climate vulnerability index is novelty of the paper. Describe adequately as many readers are not aware of it.

Response: Thank you for your suggestion. While reviewer thinks that vulnerability index is the novelty of the paper, we have a different opinion as the vulnerability index is already published in a separate form and this paper only extends the analysis by studying the impact of vulnerability of agriculture to climate change. Nevertheless, as suggested, we have revised the methods section to provide more details about the climate vulnerability index.

5. Give a two way graph of vulnerability index with mean years of schooling (nfhs 4) or with Dist Devl Index by Mohanty et al to check its association with devl.

Response: We feel the linkages between climate vulnerability and development do not align with study’s objective. Also, the rationale of two-way graph with mean years of schooling is not clear. Therefore, we have not added the suggested graph.

6. Describe detail of variables in table S1

Response: Thank you for the suggestion. We have tried to elaborate the indicator definitions. We have also moved Table S1 to the manuscript as Table 1.

7. Figure S1 and a map on stunting at dt level map be beginning. Also give correlation coeffcient of these two

Response: We appreciate the suggestion on including a supplementary map on prevalence of stunting at the district level. Since the district level information on stunting (per NFHS) and the relevant maps on stunting are easily available elsewhere we have decided not to include it in the paper. Also, we have several other child nutrition indicators in this paper; hence, giving map only on stunting may not add any further value.

8. Table 1: I suppose t1 has been derived after merging dist index value in data file. In such cases the index is a constant across district. I suggest table 1 may be presented as dt level analyses and dt is the unit of analyses. It may be appropriate to do so

Response: Yes, Table 1 (Now Table 2 in revised version) was derived by combining district level index with child level data. Since the outcome in this case is child nutrition, which is available at the child level, it is more appropriate to undertake the analysis at child level so that the socio-demographic differentials related to the outcome can be captured accurately.

9. Mixed effect model, can you show the VPC?

Response: As suggested, we have included the intra-class correlation coefficient in the revised manuscript.

10.T2 is univariate moran I?

Response: Yes, it is univariate.

11. How classification of very low----- very high of vulnerability index made

Response: All the Indian districts were categorized into five equal quintiles. The bottom 20% were considered to have very low vulnerability and the top 20% were considered to have very high vulnerability.

12. Authors may consider reduction in length of title

Response: We think the title is appropriate to reflect the essence of the paper. Therefore, we have refrained from changing the title.

We again thank the reviewers and you for giving us this opportunity and will be happy to provide further clarification if required.

With warm regards

Bidhu

[bbmahaptra@gmail.com](mailto:bbmahaptra@gmail.com)
